# Artificial Intelligence and Machine Learning in Sensing and Image Processing

**DOI:** 10.3390/s25061870

**Published:** 2025-03-18

**Authors:** Jing Chen, Miaohui Wang, Chih-Hsien Hsia

**Affiliations:** 1 Institute of Information Science and Engineering, Huaqiao University, Quanzhou 362021, China; 2 School of Information Engineering, Shenzhen University, Shenzhen 518052, China; wang.miaohui@gmail.com; 3 Department of Computer Science and Information Engineering, National Ilan University, Yilan City 260, Taiwan; hsiach@niu.edu.tw

## 1. Introduction

With the explosive growth of visual data, image sensor technology has continuously advanced [1], significantly improving resolution, dynamic range, and sampling speed while integrating more powerful processing capabilities and larger memory capacity [2]. This enables devices such as smartphones, cars, and computers to efficiently process images and video locally, reducing dependence on remote servers, thereby enhancing processing speed as well as privacy and data security [3]. The rapid development of image sensors provides artificial intelligence (AI) and machine learning (ML) with richer and higher-quality data, driving their widespread application in image processing [4].

Advancements in AI and ML in the field of vision enable machines not only to simulate human vision but also to perform more complex image analysis tasks. Compared to traditional methods that rely on manually designed algorithms [5,6], deep learning technologies enhance image recognition accuracy and flexibility through big data learning. In various fields, computer vision systems have surpassed the limitations of traditional algorithms, demonstrating superior performance in tasks such as object detection [7,8], semantic segmentation [9,10], and image enhancement [11,12]. These technological breakthroughs have been widely applied in key areas such as autonomous driving [13,14], medical image analysis [15,16], satellite remote sensing [17,18], intelligent security surveillance [19], and smart agriculture [20], accelerating digital transformation and providing crucial support for innovation across industries.The advancements of AI and ML in perception and image processing are driving industry transformation. In-depth research and enhancement of their applications will promote technological development and support broader practical applications.

This Special Issue brings together promising research in artificial intelligence and machine learning within the field of image processing, showcasing innovations and advancements of these technologies across various application scenarios. A total of thirteen papers have been accepted, covering several key areas in image processing, including image watermark embedding, femur health classification, liver tumor segmentation, X-ray data augmentation, image super-resolution, check amount recognition, automatic modulation classification, surface defect recognition, pedestrian re-identification, object detection, image de-raining, and facial image transformation synthesis. The following is a brief introduction to each paper in this special issue, highlighting their significant contributions to advancing the development of image processing technologies.

## 2. Overview of Contributions

Contribution 1 proposed an image watermarking scheme that embedded watermarks into the DWT-DCT composite transformed coefficients, effectively resisting common image processing operations and geometric attacks. To enhance robustness against scaling attacks, a resampling detection network was designed to automatically detect the scaling factor and rescale the scaled image prior to watermark detection. Additionally, to improve robustness against cutting attacks, a template watermark was embedded in the Y channel to locate the cropping region. Experimental results demonstrated that the proposed scheme achieved superior performance in both imperceptibility and robustness.

Contribution 2 introduced a femur health classification method based on geometric features and texture analysis, aiming to distinguish between healthy and unhealthy femurs and identify key features. The study used proximal femur (PFB) data from 284 Iranian cases, combining DEXA scans and MRI images, and extracted 204 geometric and texture features. Classification algorithms such as SVM, decision trees, and logistic regression were applied, with feature selection optimized using a genetic algorithm (GA). The results showed that the SVM with a radial basis function kernel performed best (89.08%), with geometric features being the most influential in classification. This study innovatively combined MRI and DEXA scans, providing a new machine learning method for femoral classification.

Contribution 3 presented a deep learning-based method for liver tumor segmentation and liver organ recognition, addressing the issue of liver and liver tumor recognition in computed tomography (CT) images. This method, based on the LiTS17 database, employed four Chebyshev graph convolution layers and one fully connected layer, enabling efficient and accurate segmentation of the liver and liver tumors. Experimental results demonstrated that the method performed excellently across various metrics, including accuracy (99.1%), Dice coefficient (91.1%), mean IoU (90.8%), sensitivity (99.4%), precision (99.4%), and recall (91.2%). Additionally, the robustness of the method in noisy environments was validated, with liver organ segmentation accuracy remaining around 90% at SNR = −4 dB. Compared to existing methods, this model showed significant performance improvements, indicating its potential for clinical diagnostic applications.

Contribution 4 provided an innovative data augmentation method aimed at addressing the issue of insufficient X-ray training data. The method generated synthetic X-ray images for training semantic segmentation models by combining X-ray images of nuclear items with cargo background images. To validate the effectiveness of this approach, the researchers trained multiple representative semantic segmentation models and conducted extensive quantitative and qualitative evaluations. Experimental results showed that the proposed augmentation method significantly enhanced the performance of segmentation models, particularly in response to item insertions and occlusion expressions in actual X-ray cargo inspection scenarios. This research provided significant support for the development of automated cargo inspection technologies, with important applications in preventing the illegal transfer of nuclear items.

Contribution 5 proposed an innovative cascaded degradation-aware blind super-resolution network (CDASRN) to address the robustness issue faced by traditional image super-resolution methods when the degradation model did not match the actual degradation in real-world scenarios. The network improved super-resolution reconstruction accuracy by eliminating the impact of noise on blur kernel estimation and estimating spatially varying blur kernels. Additionally, the introduced contrastive learning mechanism enabled CDASRN to effectively distinguish subtle differences between local blur kernels, significantly enhancing its performance in practical applications. Experimental results showed that CDASRN outperformed existing state-of-the-art methods on both severely degraded synthetic datasets and real-world datasets, demonstrating its strong robustness and wide applicability under multiple degradation factors.

Contribution 6 developed an innovative end-to-end system for recognizing courtesy amounts from Arabic check images. The system addressed the unique challenges in Arabic check amount recognition by combining rule-based modules with machine learning modules. Unlike traditional isolated digit recognition tasks, amount recognition on checks involves complex image processing and multi-layered pattern recognition, requiring specific processing methods. The system conducted an in-depth study and comparison of segmentation-based and segmentation-free approaches, offering an effective solution. Evaluation results showed that the system performed excellently on the CENPARMI dataset, achieving 67.4% accuracy at the amount level and 87.15% accuracy at the digit level, providing a valuable benchmark for future Arabic check courtesy amount recognition research.

Contribution 7 introduced an innovative lightweight neural network-based automatic modulation classification (AMC) framework to address the challenges of deploying neural networks in scenarios with strict low-latency and storage requirements. The framework enhanced classification performance by combining complex convolution with residual networks. To achieve a lightweight design, depthwise separable convolution was employed, significantly reducing the number of parameters and computational complexity. To counteract any potential performance loss from the lightweight design, a hybrid data augmentation scheme was implemented, optimizing data input to further improve the model’s performance. Simulation results demonstrated that the framework reduced the number of parameters by approximately 83.34% and the FLOPs by around 83.77%, all while maintaining performance and significantly reducing computational burden, showcasing remarkable efficiency and effectiveness.

Contribution 8 addressed the issue of insufficient generalization in surface defect recognition by proposing an image-to-image translation method based on Generative Adversarial Networks (GANs) with fine-grained labels. The method introduced a GAN model called Magna-Defect-GAN, which controlled the image generation process and produced high-quality defect images with significant intraclass variation. Surface defect data was first obtained using Magnetic Particle Inspection (MPI), then new synthetic images were generated using Magna-Defect-GAN. The expanded dataset was subsequently used to train a defect recognition model. Experiments showed that Magna-Defect-GAN could generate high-resolution, realistic defect images and significantly improved recognition accuracy. The method demonstrated good adaptability and could be integrated with other recognition models.

Contribution 9 introduced an innovative margin-based modality adaptive learning (MMAL) method specifically designed for visible-infrared person re-identification (VIPR) tasks. The method effectively improved person re-identification performance by learning appearance-discriminative features through the use of triplet loss and label-smoothed cross-entropy function in each domain. To avoid excessive suppression of modal differences, a margin-based maximum mean discrepancy (M3D) loss function was designed to ensure that discriminative features were preserved in each domain. The MMAL method enhanced the matching between visible and infrared domains by learning modality-invariant and distinctive features. On the RegDB dataset, the MMAL method achieved excellent results with a Rank-1 accuracy of 93.24% and an average precision of 83.77% in the visible-to-infrared retrieval mode, demonstrating its state-of-the-art performance in VIPR tasks.

Contribution 10 proposed a pedestrian re-identification model that combines cross-consistency learning and multi-feature fusion to address the impact of occlusion and lighting on feature extraction. The model incorporates attention mechanisms and mixed pooling modules into a residual network to automatically focus on key features in pedestrian images. The dataset is divided by camera view and classifiers are trained separately for each view, extracting view-invariant features to reduce the effects of view, pose, and background variations. A feature pyramid fuses multi-level features, further enhancing key information extraction. The model is optimized by combining cosine Softmax loss, triplet loss, and cluster center loss. Experimental results show that the model achieved 95.9% and 89.7% accuracy on the Market-1501 and DukeMTMC-reID datasets, respectively, demonstrating excellent feature extraction capabilities.

Contribution 11 designed a feature-enhancement and channel-attention-guided single-shot detector, FCSSD, aimed at addressing the multi-scale challenges in object detection. The model enhances detection performance through four key modules. First, an efficient feature extraction module (EFM) is designed to explore spatial contextual information. Then, a pyramidal aggregation module (PAM) is introduced to reduce the semantic gap between multi-scale features. Next, a feature pyramid refinement fusion (FPRF) module is constructed to further refine the features. Finally, an attention-guided module (AGM) is incorporated to optimize the fused features, alleviating the aliasing effects of FPN while maintaining a low computational burden. FCSSD combines shallow and deep-layer information, achieving a good balance in multi-scale object detection. Experimental results show that it performs comparably to mainstream methods on the PASCAL VOC and MS COCO datasets.

Contribution 12 developed a new Scale-space Feature Recalibration Network (SFR-Net) for single image deraining. The network enhances the Multi-scale Extraction Recalibration Block (MERB) by using dilated convolutions with different kernel sizes, effectively extracting and characterizing multi-scale rain streak features. Additionally, a Subspace Coordinated Attention Mechanism (SCAM) was designed and embedded into MERB, combining coordinated attention recalibration with a subspace attention mechanism to accurately adjust feature information, remove redundancy, and enhance the transfer of important features. The network structure employs dense connections and cross-layer feature fusion, repeatedly utilizing feature maps to enhance the network’s understanding and avoid the vanishing gradient problem. Through extensive experiments, SFR-Net outperforms existing state-of-the-art algorithms in terms of rain removal and detail preservation.

Contribution 13 provided an unsupervised model to address the issues of missing key facial details and lack of realism in face sketch-photo synthesis. The model is built on the CycleGAN architecture and uses a multi-scale feature extraction module to preserve semantic information in the target domain. Additionally, a convolutional block attention module (CBAM) is introduced into the generator to enhance the model’s ability to extract important features. Through CBAM, the model effectively improves the quality of the converted image and reduces artifacts caused by background interference. Furthermore, a multi-level cycle consistency loss function is constructed to ensure that the generated photo retains more identity information. Both qualitative and quantitative experimental results show that the proposed method produces clearer and more realistic facial details and edge structures in the synthesis process, and outperforms existing methods in performance metrics such as structural similarity and peak signal-to-noise ratio.

## 3. Conclusions

This Special Issue gathers significant research achievements in artificial intelligence and machine learning in the field of image processing, covering several cutting-edge applications and technological advancements. These studies not only offer intelligent solutions to current challenges but also open up new prospects for applications in image processing. We believe these contributions will provide valuable insights for researchers and practitioners, driving continued innovation in both academia and industry, and advancing image processing technology in terms of accuracy, efficiency, and practicality.

## Data Availability

The related datasets can be referred to each contribution in this Editorial.
